# Evaluation of the transporter-mediated herb-drug interaction potential of DA-9801, a standardized dioscorea extract for diabetic neuropathy, in human *in vitro* and rat *in vivo*

**DOI:** 10.1186/1472-6882-14-251

**Published:** 2014-07-17

**Authors:** Im-Sook Song, Tae Yeon Kong, Hyeon-Uk Jeong, Eun Nam Kim, Soon-Sang Kwon, Hee Eun Kang, Sang-Zin Choi, Miwon Son, Hye Suk Lee

**Affiliations:** 1 College of Pharmacy and Research Institute of Pharmaceutical Sciences, Kyungpook National University, Daegu 702-701, Korea; 2 College of Pharmacy, The Catholic University of Korea, Bucheon 420-743, Korea; 3 Research Center, Dong-A ST Co., Ltd., Yongin 446-905, Korea

**Keywords:** Transporter-mediated herb-drug interaction, DA-9801, OCT1, OCT2, OAT3, Cimetidine, Furosemide

## Abstract

**Background:**

Drug transporters play important roles in the absorption, distribution, and elimination of drugs and thereby, modulate drug efficacy and toxicity. With a growing use of poly pharmacy, concurrent administration of herbal extracts that modulate transporter activities with drugs can cause serious adverse reactions. Therefore, prediction and evaluation of drug-drug interaction potential is important in the clinic and in the drug development process. DA-9801, comprising a mixed extract of *Dioscoreae rhizoma* and *Dioscorea nipponica* Makino, is a new standardized extract currently being evaluated for diabetic peripheral neuropathy in a phase II clinical study.

**Method:**

The inhibitory effects of DA-9801 on the transport functions of organic cation transporter (OCT)1, OCT2, organic anion transporter (OAT)1, OAT3, organic anion transporting polypeptide (OATP)1B1, OATP1B3, P-glycoprotein (P-gp), and breast cancer resistance protein (BCRP) were investigated in HEK293 or LLC-PK1 cells. The effects of DA-9801 on the pharmacokinetics of relevant substrate drugs of these transporters were also examined *in vivo* in rats.

**Results:**

DA-9801 inhibited the *in vitro* transport activities of OCT1, OCT2, OAT3, and OATP1B1, with IC_50_ values of 106, 174, 48.1, and 273 μg/mL, respectively, while the other transporters were not inhibited by 300 μg/mL DA-9801. To investigate whether this inhibitory effect of DA-9801 on OCT1, OCT2, and OAT3 could change the pharmacokinetics of their substrates *in vivo*, we measured the pharmacokinetics of cimetidine, a substrate for OCT1, OCT2, and OAT3, and of furosemide, a substrate for OAT1 and OAT3, by co-administration of DA-9801 at a single oral dose of 1,000 mg/kg. Pre-dose of DA-9801 5 min or 2 h prior to cimetidine administration decreased the C_max_ of cimetidine in rats. However, DA-9801 did not affect the elimination parameters such as half-life, clearance, or amount excreted in the urine, suggesting that it did not inhibit elimination process of cimetidine, which is governed by OCT1, OCT2, and OAT3. Moreover, DA-9801 did not affect the pharmacokinetic characteristics of furosemide, as evidenced by its unchanged pharmacokinetic parameters.

**Conclusion:**

Inhibitory effects of DA-9801 on OCT1, OCT2, and OAT3 observed *in vitro* may not necessarily translate into *in vivo* herb-drug interactions in rats even at its maximum effective dose.

## Background

Diabetic peripheral neuropathy is one of the most debilitating complications of type 1 and type 2 diabetes and its histopathology is characterized by axonal degeneration, demyelination, and atrophy [1,2]. Approximately 50% of diabetes patients have symptoms of diabetic peripheral neuropathy [3]. The pursuit of new drugs for the treatment of diabetic peripheral neuropathy has led to the identification of DA-9801, an ethanol extract of *Dioscoreae rhizoma* and *Dioscorea nipponica* Makino, as a potential therapeutic agent; it is currently being evaluated in a phase II diabetic neuropathy clinical study in Korea [4]. DA-9801 may improve diabetic neuropathy-induced tissue damage by increasing nerve growth factor levels in target tissues, improving nerve conduction velocity, and promoting recovery from neuronal degeneration [4,5]. It also showed neuroprotective effects on peripheral nerves in streptozotocin-induced diabetic rats [6,7].

Herb-drug interactions, resulting from concurrent use of herbal drugs may cause adverse reactions such as toxicity and treatment failure [8]. The mechanisms underlying herb-drug interactions involve inhibition or induction of cytochrome P450 (CYP) enzymes, UDP-glucuronosyltransferase (UGT) enzymes, and drug transporters [9,10]. St. John’s wort (*Hypericum perforatum*), ginkgo (*Ginko biloba*), ginseng (*Panax ginseng*), milk thistle (*Silybum marianum*), and licorice (*Glycyrrhiza glabra*) have been reported to cause drug interactions with anticoagulants, antiretroviral drugs, anticancer drugs, immunosuppressants, or antidepressants [11-15]. Therefore, it is necessary to evaluate herb-drug interactions in order to prevent potentially dangerous clinical outcomes. DA-9801 did not potently inhibit CYP 1A2, 2A6, 2B6, 2C8, 2C9, 2C19, 2D6, or 3A4 and UGT 1A1, 1A4, 1A9, or 2B7 in human liver microsomes, indicating that DA-9801 may not inhibit the metabolism of CYP- and UGT-catalyzed drugs in humans [8].

In this study, we investigated possible herb-drug interactions involving drug transporters by using HEK293 and LLC-PK1 cell systems overexpressing clinically important uptake and efflux transporters such as organic cation transporter (OCT) 1, OCT2, organic anion transporter (OAT) 1, OAT3, organic anion transporting polypeptide (OATP) 1B1, OATP1B3, P-glycoprotein (P-gp or MDR1), and breast cancer resistance protein (BCRP) [16]. We also investigated the effects of DA-9801 on the pharmacokinetics of substrates for the effected transporters *in vivo* in rats.

## Methods

### Chemicals and reagents

Dried *Dioscoreae rhizoma* and rhizome of *Dioscorea nipponica* Makino were purchased at a speciality market for traditional herbal medicine (Dong Kyung Pharm. Co., Seoul, Korea) and their identity was kindly confirmed by Prof. Yeong Bae Seo (a specialist in plant classification, Natural Products Research Institute, Seoul National University, Seoul, Korea). The voucher specimens for *Dioscoreae rhizoma* (deposit code, KNJS) and rhizome of *Dioscorea nipponica* Makino (deposit code, LY026) were deposited at Dong-A ST Research Center (Youngin, Korea).

DA-9801 was prepared as previously reported [5]. Briefly, dried *Dioscoreae rhizoma* and rhizome of *Dioscorea nipponica* Makino were mixed in a specific ratio (3.5:1) and extracted with 50% ethanol three times at room temperature for 48 h. After filtration, the aqueous ethanol extract was evaporated under reduced pressure and lyophilized to completely remove the residual solvent and to yield brown powder. The levels of two marker components - dioscin (1.37%) and allantoin (3.29%) - in DA-9801 were determined using high performance liquid chromatography [5].

[^3^H]Methyl-4-phenylpyridinium (MPP^+^, 2.9 TBq/mmol), [^3^H]para*-*aminohippuric acid (PAH, 0.13 TBq/mmol), [^3^H]estrone-3-sulfate (ES, 2.12 TBq/mmol), [^3^H]digoxin (1.103 TBq/mmol) and [^3^H]estradiol-17β-D-glucuronide (EG, 2.22 TBq/mmol) were purchased from Perkin Elmer Inc. (Boston, MA, USA). Cimetidine, furosemide and tiapride were obtained from Sigma-Aldrich Co. (St. Louis, MO, USA). 4-Hydroxydiclofenac-*d*_4_ was obtained from Toronto Research Chemicals Inc. (North York, Ontario, Canada). All other chemicals were reagent grade and all solvents were HPLC grade.

### Inhibitory effects of DA-9801 on transport activities

HEK293 cells transiently overexpressing OAT1, OAT3, OCT1, OCT2, OATP1B1, and OATP1B3 transporters were purchased from Corning-Gentest (Tewksbury, MA, USA). The cells were maintained at 37°C in a humidified atmosphere of 8% CO_2_, in Dulbecco’s modified Eagle’s medium supplemented with 10% fetal bovine serum, 5 mM non-essential amino acids, and 100 U/mL penicillin–streptomycin. For experiments, 10^5^ cells were seeded in 96-well plates. After 24 h, the growth media were discarded and the attached cells were washed with Hank’s balanced salt solution (HBSS) and preincubated for 20 min in HBSS at 37°C. To examine the effects of DA-9801 on transporter activity, the uptake of 0.1 μM [^3^H]MPP^+^ for OCT1 and OCT2, 1 μM [^3^H]PAH for OAT1, 0.1 μM [^3^H]ES for OAT3 and OATP1B1, and 0.1 μM [^3^H]EG for OATP1B3 was measured in the presence of DA-9801 (1–300 μg/mL) for 10 min at 37°C. The cells were then washed three times with 100 μL of ice-cold HBSS immediately after placing the plates on ice and lysed with 10% SDS. The radioactivity of the probe substrate in the cells was measured using a liquid scintillation counter.

LLC-PK1-MDR1 (LLC-PK1 cells stably expressing P-gp; purchased from Corning-Gentest) and LLC-PK1-BCRP (LLC-PK1 cells stably expressing BCRP; obtained from Dr. A.H. Schinkel, Netherlands Cancer Institute, Amsterdam, The Netherlands) cells were used for the comparison of the basal to apical (B to A) transport rate of [^3^H]digoxin and [^3^H]ES in the absence and presence of DA-9801. Briefly, the cells were seeded on filter inserts for 24-transwell plates at a density of 5 × 10^5^ cells and grown for 5 days. The integrity of the cell monolayers was evaluated prior to transport experiments by measuring transepithelial electrical resistance (TEER) and TEER values in the range of 300–850 Ω · cm^2^ were used in the transport experiment [17]. For measurement of B to A transport, 0.8 mL of HBSS containing [^3^H]digoxin or [^3^H]ES (0.1 μM each) and DA-9801 (1–300 μg/mL) was added on the basal side, and 0.3 mL of fresh HBSS was added on the apical side. At every 15 min, 0.2 mL of HBSS sample in the apical side was removed and replaced with 0.2 mL of fresh HBSS for 1 h.

### Effect of DA-9801 pretreatment on the pharmacokinetics of cimetidine and furosemide in rats

Sprague–Dawley (SD) rats (male, 7 weeks old) were obtained from Samtako Co. (Osan, Korea). Animals were acclimated for 1 week in a temperature controlled room (23 ± 2°C), with a relative humidity of 55 ± 10%, an illumination intensity of 150–300 lux, a frequency of air ventilation of 15–20 times/h, and a 12 h illumination (07:00–19:00). Food and water were supplied *ad libitum*. All animal procedures were approved by the Animal Care and Use Committee in The Catholic University of Korea.

Rats were cannulated with polyethylene tubing (PE-50, Natsume Co, Tokyo, Japan) in the jugular vein for sampling under anesthesia with isoflurane. Each rat was housed individually in a rat metabolic cage and allowed to recover from anesthesia. The rats were not restrained at any time during the study. Heparinized isotonic saline (10 U/mL) was used to flush the catheters to prevent blood clotting. The rats were fasted for more than 12 h before oral administration of drugs.

DA-9801 was dissolved in DMSO/propylene glycol/deionized water (2:6:2, v/v), and a 1,000 mg/kg dose was administered to the rats by oral gavages (vehicle dosing volume, 3 mL/kg) at 5 min and 2 h prior to the oral administration of cimetidine at a dose of 10 mg/kg [18] and furosemide at a dose of 10 mg/kg [19] (vehicle dosing volume, 2 mL/kg). Cimetidine and furosemide were dissolved in deionized water and DMSO/propylene glycol/deionized water (2:4:4, v/v/v), respectively. Blood samples were collected prior to cimetidine and furosemide administration (to serve as a control), 5, 15, 30, 45 min, and 1, 1.5, 2, 3, 4, 6, 8 h after oral administration of cimetidine and furosemide. After centrifugation of blood samples at 13,000 rpm for 5 min, plasma samples (30 μL) were collected and stored at -80°C until analysis. At 24 h after drug administration, the metabolic cage was rinsed with distilled water (10 mL), and the rinsed solutions were combined with the pooled urine samples collected for 24 h. After measuring the exact volume of the urine samples, 30 μL aliquots of each sample were stored at -20°C until analysis.

### LC-MS/MS analysis of cimetidine

The concentrations of cimetidine were analyzed using a modified liquid chromatography tandem mass spectrometry (LC-MS/MS) method reported by Sun et al. [20]. Thirty μL of rat plasma samples, calibration standards, and quality control (QC) samples were vortex-mixed with 100 μL of tiapride in methanol (5 ng/mL, internal standard, IS) for 3 min at a high speed. After centrifugation at 13,000 rpm at 4°C for 8 min, 50 μL of the supernatant was diluted with 50 μL of water. An aliquot (3 μL) was injected into the LC-MS/MS system. Ten μL of rat urine samples, urine calibration standards, and QC samples were vortex-mixed with 1,000 μL of tiapride in methanol (5 μg/mL) for 3 min at high speed. After centrifugation at 13,000 rpm at 4°C for 8 min, 10 μL of the supernatant was diluted with 200 μL of 30% methanol and an aliquot (3 μL) was injected into the LC-MS/MS system. Plasma calibration standards were 1–1000 ng/mL and urine calibration standards were 1–200 μg/mL.

The LC-MS/MS system consisted of an Agilent 1200 series (Agilent Technologies, Wilmington, DE, USA) and a 6460 triple quadrupole mass spectrometer (Agilent Technologies). Mass Hunter software (Agilent Technologies) was used for LC-MS/MS system control and data processing. Separation was performed on a Luna phenyl-hexyl column (5 μm, 2.1 mm i.d. × 100 mm, Phenomenex, Torrance, CA, USA) with a gradient elution of 5% methanol with 10 mM ammonium formate (mobile phase A) and 95% methanol with 10 mM ammonium formate (mobile phase B) at a flow rate of 0.4 mL/min: 30% mobile phase B for 0.5 min, 30% to 85% mobile phase B for 0.5 min, 85% mobile phase B for 3.0 min, 85% to 30% mobile phase B for 0.1 min, 30% mobile phase B for 4 min. The column and autosampler were maintained at 50°C and 5°C, respectively. Electrospray ionization (ESI) source settings for ionization of cimetidine in the positive mode were as follows: gas temperature, 350°C; gas flow, 10 L/min; nebulizer, 35 psi; sheath gas temperature, 330°C; sheath gas flow, 11 L/min, and capillary voltage, 3500 V. Fragmentation of molecular ions for cimetidine and tiapride was performed at a collision energy of 10 eV and 18 eV, respectively. Selected reaction monitoring (SRM) mode was used for quantification: *m/z* 253.1 → 159.1 for cimetidine and *m/z* 329.1 → 256 for tiapride.

### LC-MS/MS analysis of furosemide

The concentrations of furosemide were analyzed using a modified LC-MS/MS method reported by Sora et al. [21]. Thirty μL of rat plasma samples, calibration standards, and QC samples were vortex-mixed with 100 μL of 4-hydroxydiclofenac-*d*_4_ in methanol (500 ng/mL, IS) for 3 min at a high speed. After centrifugation at 13,000 rpm at 4°C for 8 min, 60 μL of the supernatant was diluted with 40 μL of water. An aliquot (5 μL) was injected into the LC-MS/MS. Ten μL of rat urine samples, calibration standards, and QC samples were vortex-mixed with 1000 μL of 4-hydroxydiclofenac-*d*_4_ in methanol (15 μg/mL) for 3 min at high speed. After centrifugation at 13,000 rpm at 4°C for 8 min, the aliquot (5 μL) was injected into the LC-MS/MS. Plasma calibration standards were 0.02-20 μg/mL and urine calibration standards were 1–200 μg/mL. Separation was performed on a Pinnacle DB Biphenyl column (3 μm, 2.1 mm i.d. × 50 mm, RESTEK, USA) using gradient elution of 5% methanol in 0.1% formic acid (mobile phase A) and 95% methanol in 0.1% formic acid (mobile phase B) at a flow rate of 0.3 mL/min: 50% mobile phase B for 0.4 min, 50% to 95% mobile phase B for 0.1 min, 95% mobile phase B for 4.0 min, 95% to 50% mobile phase B for 0.1 min, 50% mobile phase B for 4.0 min. The column and autosampler were maintained at 50°C and 5°C, respectively. ESI source settings for ionization of furosemide and IS in the negative mode were as follows: gas temperature, 350°C; gas flow, 10 L/min; nebulizer, 35 psi; sheath gas temperature, 350°C; sheath gas flow, 11 L/min, and capillary voltage, 3500 V. Fragmentation of molecular ions for furosemide and 4-hydroxydiclofenac-*d*_4_ was performed at a collision energy of 7 eV and 4 eV, respectively. SRM mode was used for quantification: *m/z* 329.1 → 284.9 for furosemide and *m/z* 315.1 → 270.9 for 4-hydroxydiclofenac-*d*_4_.

### Pharmacokinetic and statistical analyses

In the inhibition studies, the percentages of inhibition were calculated using the ratio of the transport rate of probe substrates with or without DA-9801 and the relevant data were fitted to an inhibitory effect model [i.e. $v=E_{\text{max}}\left(1-\frac{\left[I\right]}{IC_{50}+\left[I\right]}\right)$] [22]. Calculations were performed using WinNonlin software (ver. 2.0, Pharsight, Mountain View, LA).

In the *in vivo* rat studies, non-compartmental pharmacokinetic analysis was also performed using the WinNonlin software. The area under the plasma concentration–time curve (AUC) was calculated using the linear trapezoidal method. The area from the last datum point to time infinity (AUC_∞_) was estimated by dividing the last measured concentration in plasma by the terminal rate constant. The terminal elimination half-life (t_1/2_) and the systemic clearance (CL/F) were determined.

Statistical significance was analyzed using the Mann–Whitney U test, and values of *p* < 0.05 were considered statistically significant. The SPSS software package (ver. 19.0, SPSS, Chicago, IL) was used for statistical analysis.

## Results

### Inhibitory effect of DA-9801 on the activities of drug transporters

**Figure 1:**
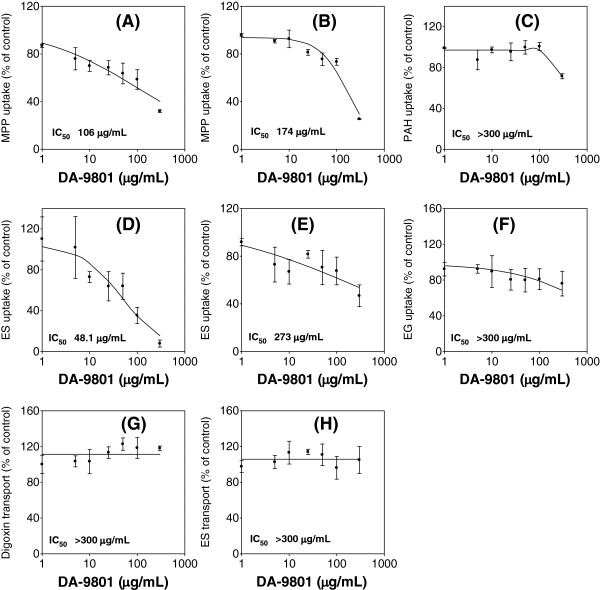
**Inhibitory effect of DA-9801 on the transport activities of OCT1 (A), OCT2 (B), OAT1 (C), OAT3 (D), OATP1B1 (E), OATP1B3 (F), P-gp (G), and BCRP (H).** Inhibitory effect of DA-9801 (1–300 μg/ml) on the uptake of 0.1 μM [^3^H]methyl-4-phenylpyridinium (MPP^+^; a substrate for OCT1 and OCT2), 1 μM [^14^C]para*-*aminohippuric acid (PAH; a substrate for OAT1), 0.1 μM [^3^H]estrone-3-sulfate (ES; a substrate for OAT3, OATP1B1, and BCRP), 0.1 μM [^3^H]estradiol-17β-D-glucuronide (EG; a substrate for OATP1B3), and 0.1 μM [^3^H]digoxin (a substrate for P-gp) were measured. Data point represents the mean ± SD of three independent experiments. Data were fitted to an inhibitory effect E_max_ model and the IC_50_ value was calculated.

To characterize the inhibitory effect of DA-9801 on the uptake transporters, we measured the uptake of representative substrates for each transporter in HEK293 cells overexpressing OAT1, OAT3, OCT1, OCT2, OATP1B1, and OATP1B3 in the presence of DA-9801 (1–300 μg/mL). DA-9801 inhibited the transport activity of OCT1, OCT2, OAT3, and OATP1B1 with IC_50_ values of 106, 174, 48.1, and 273 μg/mL, respectively (Figure 1A, B, D, and E). However, DA-9801 did not inhibit 50% of the transport activity of OAT1 and OATP1B3 at its highest concentration used in this study (300 μg/mL). Therefore, we could not calculate IC_50_ values for these transporters (Figure 1C and F). We further examined the inhibitory effect of DA-9801 on P-gp (MDR1) and BCRP function, by examining its effect on the B to A transport rate of digoxin and ES in LLC-PK1-MDR1 cells and LLC-PK1-BCRP cells, respectively. Efflux of digoxin and ES via P-gp and BCRP, respectively, was not inhibited by the presence of DA-9801 at concentrations up to 300 μg/mL (Figure 1G and H).

### Effects of DA-9801 on the pharmacokinetics of cimetidine and furosemide in rats

To assess the relevance of the DA-9801 IC_50_ values obtained *in vitro* to the *in vivo* DA-9801 herb-drug interaction with substrates for OCT1, OCT2, and/or OAT3, cimetidine was selected as a substrate for OCT1, OCT2 and OAT3 [23] and furosemide for OAT3 [24]. DA-9801 was orally administered 5 min and 2 h prior to the administration of cimetidine or furosemide.

**Figure 2:**
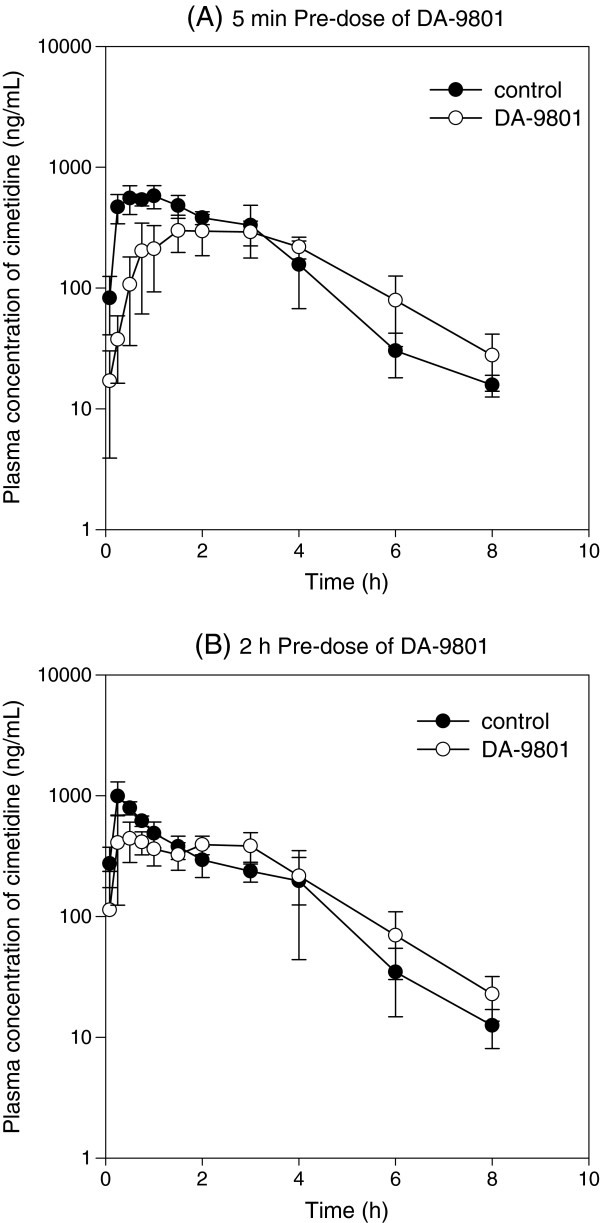
**Plasma concentration-time curves of cimetidine. (A)** Plasma concentration profile of cimetidine in rats after oral administration of 10 mg/kg cimetidine alone (●) and cimetidine with 5 min pre-dose of 1,000 mg/kg DA-9801 (○). **(B)** Plasma concentration profile of cimetidine in rats after oral administration of 10 mg/kg cimetidine alone (●) and cimetidine with 2 h pre-dose of 1,000 mg/kg DA-9801 (○). Each data point represents the mean ± S.D. of five rats.

The AUC_8h_, AUC_∞_, CL/F, and t_1/2_ of cimetidine were not changed by pre-dose of DA-9801, either at 5 min or 2 h. Consequently, the amount of cimetidine excreted in urine was not changed by the pretreatment of DA-9801. In contrast, 5 min pre-dose of DA-9801 delayed T_max_ and decreased C_max_ of cimetidine. 2 h pre-dose of DA-9801 decreased C_max_ without affecting T_max_ of cimetidine (Table 1 and Figure 2).

**Table 1:** Pharmacokinetic parameters of cimetidine (10 mg/kg) after co-administration of DA-9801 at a single oral dose of 1,000 mg/kg

	**5 min pre-dose of DA-9801 (n = 5)**		**2 h pre-dose of DA-9801 (n = 5)**	
	**Control**	**DA-9801**	**Control**	**DA-9801**
T_max_ (h)	0.700 ± 0.326	2.50 ± 0.707**	0.300 ± 0.112	1.20 ± 1.24
C_max_ (ng/mL)	652 ± 120	337 ± 100**	995 ± 305	532 ± 175*
t_1/2_ (h)	1.12 ± 0.130	1.45 ± 0.620	1.09 ± 0.139	1.20 ± 0.125
AUC_8h_ (μg/mL · h)	1.77 ± 0.314	1.34 ± 0.154	1.80 ± 0.372	1.78 ± 0.290
AUC_∞_ (μg/mL · h)	1.79 ± 0.313	1.41 ± 0.122	1.82 ± 0.382	1.82 ± 0.304
CL/F (mL/min/kg)	95.2 ± 15.7	119 ± 9.56	94.1 ± 15.5	93.5 ± 13.8
Amount excreted in urine (% of dose)	23.2 ± 4.53	16.3 ± 10.9	24.1 ± 11.2	27.4 ± 3.23

*p < 0.05, for the comparison with the control group.

**p <0.01, for the comparison with control group.

**Figure 3:**
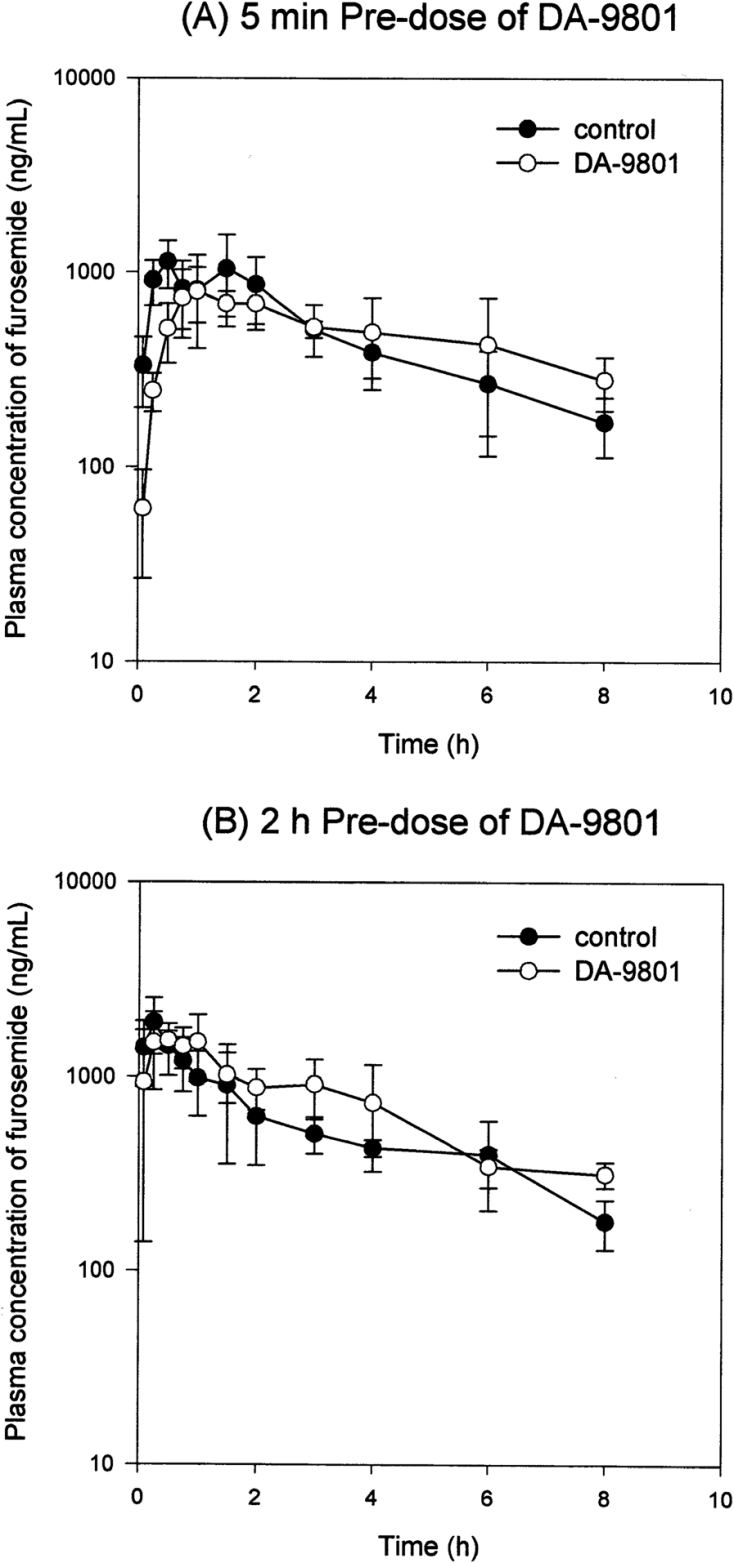
**Plasma concentration-time curves of furosemide. (A)** Plasma concentration profile of furosemide in rats after oral administration of 10 mg/kg furosemide alone (●) and furosemide with 5 min pre-dose of 1,000 mg/kg DA-9801 (○). **(B)** Plasma concentration profile of furosemide in rats after oral administration of 10 mg/kg furosemide alone (●) and furosemide with 2 h pre-dose of 1,000 mg/kg DA-9801 (○). Each data point represents the mean ± S.D. of five rats.

In contrast to these cimetidine-related observation, the pharmacokinetic parameters such as T_max_, C_max_, AUC_8h_, AUC_∞_, CL/F, t_1/2_, and the amount of furosemide excreted in urine were not affected by the co-administration of DA-9801 either 5 min or 2 h prior to furosemide (Table 2 and Figure 3).

**Table 2:** Pharmacokinetic parameters of furosemide (10 mg/kg) after co-administration of DA-9801 at a single oral dose of 1,000 mg/kg

	**5 min pre-dose of DA-9801 (n = 5)**		**2 h pre-dose of DA-9801 (n = 5)**	
	**Control**	**DA-9801**	**Control**	**DA-9801**
T_max_ (h)	0.600 ± 0.518	1.35 ± 0.602	0.250 ± 0.000	0.550 ± 0.411
C_max_ (μg/mL)	1.32 ± 0.330	0.896 ± 0.212	1.92 ± 0.619	1.98 ± 0.364
t_1/2_ (h)	3.40 ± 1.51	3.95 ± 1.09	3.40 ± 1.43	3.06 ± 0.920
AUC_8h_ (μg/mL · h)	4.00 ± 1.02	4.94 ± 2.58	4.67 ± 0.819	5.96 ± 1.11
AUC_∞_ (μg/mL · h)	4.91 ± 1.29	5.61 ± 1.09	5.59 ± 0.528	7.37 ± 1.08
CL/F (mL/min/kg)	36.7 ± 13.5	30.5 ± 5.46	30.0 ± 2.73	23.0 ± 3.72
Amount excreted in urine (% of dose)	20.2 ± 7.19	30.4 ± 14.7	17.5 ± 6.87	25.7 ± 5.70

## Discussion

The recent trend toward the use of poly pharmacy, which comprises drugs with different mechanisms of action, necessitates careful consideration of the potential drug-drug interactions (DDI) between the combined medicines. DDIs between inhibitors and substrates of metabolizing enzymes or transporters can cause serious adverse reactions. Therefore, prediction and evaluation of DDI potential is important in the clinic and in the drug development process.

It is increasingly recognized that drug transporters have a significant impact on DDIs by modulating the absorption, distribution, and excretion of drugs, alone or in interaction with drug-metabolizing enzymes [16,25,26]. In particular, screening of clinically important drug transporters such as OCT1, OCT2, OAT1, OAT3, OATP1B1, OATP1B3, P-gp, and BCRP in the drug development stage is recommended [16]. In this study, we investigated the inhibitory effects of DA-9801 on these transporters. IC_50_ values of DA-9801 for these transporters were estimated to be 106, 174, >300, 48.1, 273, >300, >300, and >300 μg/mL, respectively (Figure 1).

It is important to note that *in vivo* human studies investigating the interactions between DA-9801 and substrates for the affected transporters such as OCT1, OCT2, and OAT3 are necessary to determine whether the *in vitro* inhibition of these transporters by DA-9801 is relevant or not. The inhibition of transport activities *in vitro* can be applied to herb-drug interaction potential *in vivo* with effective highest plasma concentration, plasma free fraction, and IC_50_ values of perpetrator [16]. However, DA-9801 is a herbal extract and, this has made elucidation of a single effective component and its plasma concentration difficult. Therefore, we aimed to investigate the *in vivo* herb-drug interaction potential in rats by using DA-9801 and either cimetidine, a simultaneous substrate for OCT1, OCT2, and OAT3, or furosemide, a substrate for OAT3.

The effective dose of DA-9801 for the therapeutics of diabetic neuropathy was 100–1,000 mg/kg in rats [6,7]. Therefore, we treated DA-9801 at a dose of 1,000 mg/kg in this study since the inhibitor has been treated at a maximum effective dose to investigate the highest possibility of *in vivo* herb-drug interaction. Then, although the experimental systems and species were not perfectly matched, after determining the IC_50_ values of DA-9801 on the transport activity in HEK293 cells overexpressing human transporters such as OCT1, OCT2 and OAT3, we continued to evaluate *in vivo* herb-drug interactions between DA-9801 and substrates for Oct1, Oct2, and Oat3 in rats. Even though the tendency for the inhibitory potency of representative inhibitors in OCT and OAT transporters from different species such as mice, rat, rabbit, and humans, the IC_50_ values were different among them [27,28]. Therefore, we should note that a species difference in the transport activity between human and rat may account, in part, for the species difference in herb-drug interaction between DA-9801 and cimetidine and furosemide.

Cimetidine is a H2 receptor antagonist used for the treatment of peptic ulcers and related disorders. It is eliminated mainly by renal excretion in rats, with 70% eliminated by 72 h after oral ingestion without significant metabolism [29]. Cimetidine undergoes extensive tubular secretion in which Oct1/2 and Oat3 play major roles [23]. In case of 5 min pre-dose of DA-9801, C_max_ of cimetidine was lower and T_max_ was greater than those in the control group. DA-9801 did not affect the AUC_8h_, AUC_∞_, CL/F, and t_1/2_. With regard to 2 h pre-dose of DA-9801 prior to cimetidine, C_max_ was slightly lower than that in the control group, but there was no difference in T_max_ between treatment groups (Table 1 and Figure 2). Since average gastric emptying time in rats was about 30 min [30], differences in the C_max_ and T_max_ of cimetidine between 5 min pre-dose and 2 h pre-dose groups could be attributed to the presence and absence of DA-9801 in the stomach. After the removal of DA-9801 from the stomach (i.e. case of 2 h pre-dose of DA-9801), absorption phase of cimetidine tend to be similar to that in the control group. In addition, considering that Oat3 and Oct1/2 were mainly involved in the renal excretion of cimetidine and that CL/F and urinary excretion of cimetidine were not changed, DA-9801 (orally administered at a dose of 1,000 mg/kg) affected the intestinal absorption of cimetidine but did not modulate the elimination pathway, which is mediated by Oat3 and Oct1/2. This is supported by literature indicating that cimetidine was well absorbed after oral administration via paracellular pathway but its absorption was highly variable. Gastric pH and emptying variability have been reported to influence the absorption pattern of cimetidine as a function of dose and time of administration [31,32].

Furosemide, an inhibitor of the Na^+^–K^+^–2Cl^-^ symport that is used as loop or high-ceiling diuretic, is eliminated mainly by renal excretion in rats, with 80-90% elimination by 72 h after intravenous injection without significant metabolism [24]. Furosemide undergoes extensive tubular secretion in which Oat1/3 play major roles [24]. Neither the 5 min pre-dose nor 2 h pre-dose of DA-9801 affected the pharmacokinetic parameters of furosemide (i.e., AUC_8h_, AUC_∞_, CL/F, t_1/2_, and amount excreted in urine). Taking all these results into consideration, DA-9801, a weak inhibitor of OAT3, did not cause *in vivo* herb-drug interactions that affected the pharmacokinetics of furosemide in rats.

## Conclusions

We have investigated the inhibitory effects of DA-9801 on transport activities of clinically important transporters and explored the *in vivo* herb-drug interaction potential between DA-9801 and target transporters such as OCT1, OCT2, and OAT3 at the maximum effective dose of DA-9801 in rats (1,000 mg/kg). While DA-9801 pre-dose (5 min or 2 h) did not change the pharmacokinetics of furosemide, it decreased the C_max_ of cimetidine without changing AUC and CL/F. These results suggested that inhibitory effects of DA-9801 on OCT1, OCT2, and OAT3 transporters *in vitro* may not necessarily translate into *in vivo* herb-drug interaction in rats.

## Competing interests

The authors have declared that no competing interests exist.

## Authors’ contributions

Conceived and designed the experiments: ISS, HEK, SZC, MS, HSL. Performed the experiments: ISS, TYK, HYJ, ENK, SSK. Analyzed the data: ISS, TYK, HUJ, ENK, SSK, HEK, SZC, MS, HSL. Wrote the paper: ISS, TYK, HUJ, ENK, SSK, HEK, SZC, MS, HSL. All authors read and approved the final manuscript.

## Pre-publication history

The pre-publication history for this paper can be accessed here:

http://www.biomedcentral.com/1472-6882/14/251/prepub
